# T206. DOES AGE INFLUENCE RESPONSE TO COGNITIVE REMEDIATION?

**DOI:** 10.1093/schbul/sby016.482

**Published:** 2018-04-01

**Authors:** Benedetta Seccomandi, Deborah Agbedjro, Morris Bell, Richard Keefe, Matcheri Keshavan, Silvana Galderisi, Alice Medalia, Joanna Fiszdon, Mario Maj, Armida Mucci, Roberto Cavallato, Til Wykes, Matteo Cella

**Affiliations:** 1 King’s College London; 2 Yale University School of Medicine; 3 Duke University Medical Center; 4 Harvard University; 5 University of Naples SUN; 6 Columbia University; 7 VA Connecticut Health, Yale University; 8 University Vita-Salute San Raffaele

## Abstract

**Background:**

Cognitive deficits are common in people with schizophrenia and have a negative impact on functioning. Cognitive Remediation (CR) is an effective approach to reduce the burden of cognitive difficulties however there are individual differences in therapy response. Previous research suggests that participants age may be a significant moderator of therapy efficacy but results are inconclusive. This study attempts to fill this gap by exploring how age may influence CR outcomes.

**Methods:**

Data from ten trials from the NIMH Database of Cognitive Training and Remediation Studies (DoCTRS) were used. We considered the following therapy outcomes: Executive function as assessed by the Trail making test part B (TMTB), the Wisconsin Card Sorting Test (WCST) and Verbal fluency (FAS) scores. Working memory was assessed with the Letter-Number Span (LNS) and the Digit span. Symptoms were evaluated with the Positive, Negative and General scores from the Positive and Negative Syndrome Scale (PANSS). Functioning was assessed using the Heinrichs-Carpenter quality of life (HCQOL) scale. To evaluate the effect of age on outcomes we classified participants into under 40 and over 40 years old. We compared outcomes across age groups using mixed linear models.

**Results:**

We considered data from 711 people with schizophrenia (407 received CR and 304 the control condition). For the under 40 group the average age was 29.26 (SD 6.83) while the average yeas spent in education was 12.11 (2.61). The over 40 group had a mean age of 40.09 (SD 6.09) and 12.11 (2.54) years of education.

We found a significant interaction between age and working memory and functioning improvement for the over 40 group. The younger group showed a larger effect of CR in term of general symptoms reduction. We did not find an effect of age on executive function, positive and negative symptom.

**Discussion:**

The results indicate that CR may benefit people with schizophrenia in different way depending on their age. Age may represent a large number of complex factors and more work is needed in this area to better understand how individual characteristics and illness history may influence CR response. Work in this sense will help to reduce CR response heterogeneity and improve therapy personalisation.

